# 

**Published:** 1887-05-28

**Authors:** 


The Hospital.

An Institution, Family, and Parochial Journal of Hospitals, Asylums, and all Agencies for the Care of the Sick, Criticism and News.

SATURDAY, MAY 28TH, 1887.

### The Literary Prize.

A PRIZE of **Two Guineas** will be given every month for the best story or incident based upon hospital, or asylum, or student life, or any incident connected with the professions of medicine or nursing.

### The Children's Corner.

### Third Quarterly Prize Competition.

Began 2nd Apr., 1887 ; ends 25th June, 1887.

### Puzzles—Ninth Week.

No. 35. CONUNDRUM. Why can an omnibus never be said to be empty?

No. 36. PUZZLE SENTENCE. Write out this sentence fully: "He does not C U because he s."

No. 37. DROPPED VOWEL PUZZLE. Th bst phsens r Dr Dt Dr Qt nd Dr Mrrymn. (A proverb.)

No. 38. ENIGMA.

Two fifths of a craft, two thirds of a row,
Give the name of a bird you very well know.

### Letters—Ninth Week.

The Letter-subject for next week will be "Drawing."

### Results of Seventh Week—Puzzles.

No. 27. CONUNDRUM. Because the head is on one side and the tail on the other.

No. 28. NAMES OF TOWNS ENIGMATICALLY EXPRESSED. Hull; Wakefield; Bradford; Milton; Sunderland.

No. 29. ENIGMA. "On, Stanley, on." If you put "I" in *Stanley's place*, of course you get Onion, a thing which will "bring a tear to many an eye."

No. 30. CHARADE. A little inspection here reveals the word sought, viz., In-sat-i-ate = insatiate.

*All right*—A. C. K., Dot, Joe, Nordisa, Scrooge, Skye, Sutton, Tim, Umslopogaas; *Three right*—Alsie, Bohemian Girl, Gom, Mikado; *Two right*—A. G. S. McCulloch, Etta, Ada, The Eda; *One right*—Ellie, R. Epton, Peeler.

### Letters.

As I quite expected, all my young friends delight in books. I do not wonder at it, for they are not only our guide in youth, but our peerless companions throughout life. A man who passes his life without books, exists—he does not live. That wise man Colton has observed that a good book is to man the same as the flower is to the bee—it is that from which honey may be gathered.

Here is an interesting letter from a young friend at Glasgow:—

DEAR MR. EDITOR,—I am very fond of reading. I like the *Pickwick Papers* and all the books I have read of Charles Dickens. I am reading the *Daisy Chain* by Miss Younge; it is very nice. Mamma reads to us every Sunday evening; she is now reading the *Fairchild Family* by Mrs. Sherwood, who wrote *Stories on the Church Catechism*. I like boys' adventure books very much, such as *Gorilla Hunters*, *Coral Island*, *The Swiss Family Robinson*, and *In the Wilds of Africa*, and lots of other books. I do not like poetry so much, but *Aunt Charlotte's Evenings at Home* is very nice, and it is nearly all poetry. When I have a holiday, I generally like reading better than anything else.—I am, yours truly, THE EDA, aged 10½.
9, The College, Glasgow.

Skye tells me she could sit and read nearly all day long; fiction she prefers to history. Bohemian Girl is enamoured of *The Last of the Mohicans*. Most of Fenimore Cooper's novels deserve, I think, to be more widely read on this side of the Atlantic than they have been hitherto.

Here is a letter from a very young correspondent:—

MY DEAR MR. EDITOR,—I am afraid I do not know much about reading. I found it more difficult to learn to read than to write. I am very glad I have learnt, for I am very fond of reading. I like fairy tales very much, and I am very fond of being read to. One of my favourite books is the *Pilgrim's Progress*. I like Papa to read that to me: he told me it is a very old book.

I am, dear Mr. Editor, yours truly, ETTA, aged 8½.
Bank Buildings, Walton-on-Thames.

Peeler likes books about natural history. Ada writes a very sensible letter about reading, and the good which one can sometimes do by spending a few spare minutes to read to poor sick sufferers who cannot help themselves. Ethel, who tells me that she is known at home as the bookworm, gives me the names of a great number of interesting books which she has read.

One mark each:—Bohemian Girl, Ellie, Peeler, Skye, Ada, The Eda, Etta, Ethel.

Answers must be addressed to the "PUZZLE EDITOR," THE HOSPITAL, Norfolk House, Norfolk Street, Strand, W.C., not later than Thursday next. The "Children's Corner" Coupon (see advertisement pages) must be enclosed.

### Amusements with Profit.

### PRIZE COMPETITIONS.

### Quarterly Prize Competition.

Began 23rd April; ends 16th July.

### A Prize of Three Guineas will be awarded to the Competitor who shall have sent in the largest number of correct or best answers. No one will be permitted to win the Three Guinea Prize twice in any one year, but we shall award annually an additional

### Prize of Five Guineas to the competitor who shall have sent in the largest number of correct or best answers during the twelve months.

### No. 11. Charade.

A trisyllabic word am I,
Of letters three times three,
My *first* part gives (at least in sound)
A fish, a mat, a straight may be.
My *next*, Phœnician traders led
From their far-distant shore,
To barter here their Eastern wares,
And Albion's wealth explore.
My *third* the Bard of Avon wrote
In the title of a play.
My *whole* is a quaint punishment
Found in the East, I say.

### No. 12. Mathematical Question.

The hands of a clock coincide at 12: when will they coincide again?

### Answers.

No. 7. MATHEMATICAL QUESTION.

This is a question in Geometrical Progression. The sum of the terms=40.

$$40 = \frac{1\,(r^4 - 1)}{r - 1}$$

$$\therefore 40r - 40 = r^4 - 1$$

$$\therefore r^4 - 40r + 39 = 0$$

$$\therefore (r - 3)\,(r^3 + 3r^2 + 9r - 13) = 0$$

$$r = 3$$

The first term is 1. ∴ The weights are $1 \,.\, 3 \,.\, 3^2 \,.\, 3^3$, or 1lb., 3lb., 9lb., 27lb.

Correct answers have been received from Gretta, Broadwater, Canice, Dawnlover, Aralc, Ostrich, The Man of the Sea, Mater, Perseverance, Letheàdor, Ellice, Hexameter.

No. 8. CHARADE.

As "Newspaper" and "Lifetime" equally meet the conditions, we accept both as correct.

Correct answers have been received from Gretta, Broadwater, Canice, Dawnlover, Aralc, Ostrich, K. M., The Man of the Sea, Mater, Perseverance, May, Gip, Robin, Letheàdor, Ellice, Hexameter, A.M.E.

### The Tie.

Both Ostrich and Ellice have correctly guessed the Special Tie Acrostic:—BumP (phrenology); LeecH; AstronomY; MarS; EngedI; LaC; EnnuI; SpinozA; SamsoN. (Blameless Physician, a title bestowed by Homer on Æsculapius.) We now set the following Acrostic to decide the Tie.

### Double Acrostic.

Where they
Most breed and haunt, I have observ'd, the air
Is delicate. *Macbeth.*

A lusty winter,
Frosty but kindly. *As You Like It.*

Howbeit they would hold up this Salique law,
To bar your highness. *King Henry V.*

And am not I a prelate of the church? *King Henry VI.*

They sparkle still the right Promethean fire,
They are the books, the arts, the academes
That show, contain, and nourish all the world.
*Love's Labour's Lost.*

A slave, whose gall coins slanders like the mint.
*Troilus and Cressida.*

Haddest not thou been by,
A fellow by the hand of nature mark'd,
Quoted, and sign'd, to do a deed of shame,
This murder had not come into my mind.
*King John.*

(The initials and the finals give the names of two of Shakespeare's plays.)

Answers must be addressed to THE PRIZE EDITOR, AMUSEMENTS WITH PROFIT, THE HOSPITAL, Norfolk House, Norfolk Street, Strand, W.C., not later than the *first* post on Saturday next. The full name and address must in all cases be given, but a *nom de plume* may be added for publication. Only one side of the paper should be written on. The "Amusements with Profit" Coupon (see advertisement pages) must be enclosed with replies.

### The In- and Out-Patients' Hospital Diary.

This Diary is so arranged as to make some portion of it appear every week, and the whole in each monthly part. It has been very difficult to compile, and corrections will at all times be gratefully received. It has been suggested that similar information about the larger Provincial and Scotch Hospitals would be useful to many readers. We should be glad to hear if this is felt to be the case.

### DISEASES AND IRREGULARITIES OF THE TEETH.—*continued.*

| Hospitals and Terms of Admission. | Physicians, Surgeons, and Medical Officers, with Days and Hours of Attendance. |
|---|---|
| New Hospital for Women, 222, Marylebone rd., W. | *Surgeon*, Mr. H. Apperley |
| North-West London Hosp., 18, Kentish Town Rd., N.W. | *Surgeon*, Mr. W. A. Maggs, F. 9 |
| Paddington Green Children's Hospital, Paddington | *Surgeon*, Mr. C. Vincent Cotterell, W. 9 |
| Royal Free Hospital, Gray's Inn Road, W.C. | *Surgeon*, Mr. H. Harris, M. Th. |
| Royal Hospital for Children and Women, Waterloo Bridge Road, S.E. | *Surgeon*, Mr. Whitehouse, F. 1.30 |
| St. Bartholomew's Hospital, Smithfield, E.C. | *Surgeons*, Messrs. Ewbank, Tu.; Paterson, F.; Ackery, F.; Mackrett, Tu. Hour, 9 |
| St. George's Hospital, Hyde Park Corner, S.W. | *Surgeon*, Mr. Winterbottom, Tu. S. 9 |
| St. Mary's Hospital, Cambridge Place, Paddington | *Susgeon*, Mr. H. Hayward, W. S. 9.30 |
| St. Thomas's Hospital, Westminster Bridge Road, S.E. | *Surgeon*, Mr. Truman, Tu. F. 10 |
| University College Hospital, Gower Street, W.C. | *Surgeon*, Mr. S. J. Hutchinson, W. 9.30 |
| Victoria Hospital for Children, Chelsea, S.W. | *Surgeon*, Mr. F. Fox, S. 9.0 |
| West London Hospital, Hammersmith Road, W. | *Surgeon*, Mr. H. L. Albert, Tu. F. 9.30 |
| Westminster Hospital, Broad Sanctuary, S.W. | *Surgeons*, Messrs. J. Walker and M. A. Smale, W. S. 9.15 |

### THROAT DISEASES.

| | |
|---|---|
| Central London Throat and Ear Hospital, Gray's Inn Road, W.C. | *Surgeons. See* "Ear Cases." M. W. Th. S. 2.30; Tu. F. 5 |
| Great Northern Central Hospital, Caledonian Rd, N. | *Surgeon*, Mr. Spencer Watson, W. 2 |
| Guy's Hospital, St. Thomas Street, S.E. | *Surgeon*, Mr. Symonds, Th. 1 |
| Hospital for Diseases of the Throat and Chest, 32, Golden Sq. Hon. Sec., Mr. G. C. Witherby. Poor free; others by small payment | *Physicians*, Drs. Wolfenden, Tu. F. 2.30; Macdonald, Tu. F. 6.30; Bond, W. S. 2.30. *Surgeon*, Mr. T. Mark Hovell, M. Th. 2.30 |
| King's College Hospital, Portugal Street, W.C. | *Surgeon*, Mr. Cartwright, Tu. F. 10 |
| Middlesex Hospital, Berners Street, W. | *Surgeon*, Mr. Hensman, Tu. 9 |
| North West London Hospital, 18, Kentish Tn. Rd., N.W | *Surgeon*, Mr. Collier, M. 1.30 |
| St. Bartholomew's Hospital, Smithfield, E.C. | *Surgeon*, Mr. Butlin, F. 2.30 |
| St. George's Hospital, Hyde Park Corner, S.W. | *Surgeon*, Dr. Whipham, Th. 1.30 |
| St. Mary's Hospital, Cambridge Place, Paddington | *Surgeon*, Mr. A. T. Norton, M. Th. 9.30 |
| St. Thomas's Hospital Westminster Bridge Road, S.E. | *Physician*, Dr. Semon, Tu. F. 1.30 |
| University College Hospital, Gower Street, W.C. | *Physician*, Dr. Poore, Th. 1.30 |
| Westminster Hospital, Broad Sanctuary, S.W. | W. 9. *See* General Hospitals. |

### DISEASES OF WOMEN, (OBSTETRIC CASES, ETC.)

| | |
|---|---|
| Charing Cross Hospital, West Strand, W.C. | *Physicians*, Drs. J. W. Black, Amand Routh, Tu. F. 1.30 |
| Chelsea Hospital for Women, Fulham Road, S.W. Sec., Mr. J. H. Easterbrook. Payment from 10s. 6*d.* a week. Poor by Governor's letter. Out-patients, if without subscriber's letter, pay 6*d.* each attendance | *In-Physicians*, Drs. J. H. Aveling, M. Th.; A. W. Edis, Tu. F.; F. Barnes, W. S. Hour, 2.30. *Out-Physicians* Drs. Dickinson and Mackeron, M.; W. Travers and G. Harper, Tu.; F. Jones and J. I. Parsons, W.; and H. T. Rutherford, F. Hour, 2 |

### DISEASES OF WOMEN, (OBSTETRIC CASES, ETC.)—*Continued.*

| Hospitals and Terms of Admission. | Physicians, Surgeons, and Medical Officers, with Days and Hours of Attendance. |
|---|---|
| East London Hospital for Children and Dispensary for Women, Shadwell, E. A Subscriber's letter is required, except in urgent cases | *Out-Physicians*, M. Tu. W. Th. F. 2 and 3; Tu. F. 9. *See* "Children's Diseases." |
| German Hospital, Dalston Lane, Dalston, E. | *Physician*, Dr. Rasch, M. Th. 9 |
| Great Northern Central Hospital, Caledonian Rd., N. | *Physician*, Dr. F. Barnes, W. 2 |
| Grosvenor Hospital for Women and Children, 3 and 4, Vincent Square, S.W. | *Out-Physicians and Surgeons*, Tu. W. Th. S. 2. *See* "Children's Diseases." |
| Guy's Hospital, St. Thomas Street, S.E. | *Physicians*, Drs. Galabin, Horrocks, M. Tu. F. 1.30 |
| Hospital of St. John and St. Elizabeth, 47, Gt. Ormond Street, W.C. For females suffering from long standing disease. | *Physicians*, Drs. Constable and Tegart (no out-patients) |
| Hospital for Women, 29 and 30, Soho Sq., W. Sec., Mr. David Cannon. In-patients admitted free by Governor's letter; also by payment. Out-patients free | *In-Physicians*, Drs. Carter, W. S. 2; R. T. Smith, Tu. F. 2; E. Holland, Tu. F. 9.30. *In-Surgeon*, Mr. H. A. Reeves, M. 4, Th. 2; *Out-Physicians*, Drs. R. T. Smith, F. 10; E. Holland, W. 10; Mansell-Moullin, M. Th. 11; Bedford Fenwick, Tu. 10; J. Oliver, S. 10. *Out-Surgeon*, Mr. S. Osborn, Th. 2 |
| King's College Hospital, Portugal Street, W.C. | *Physician*, Dr. Playfair, Tu. Th. S. 2 |
| London Hospital, Whitechapel Road, E. | *In-Physician*, Dr. Herman, Tu. F. 1.30. *Out-Physician*, Dr. Lewers, W. S. 1.30 |
| Metropolitan Free Hospital, Gray's Inn Road, W.C. | *Physician*, Dr. A. Venn, W. 2 |
| Middlesex Hospital, Berners Street, W. | *In-Physician*, Dr. Edis, Tu. F. 1.30. *Out-Physician*, Dr. Duncan, W. S. 1.30 |
| New Hospital for Women, 222, Marylebone Road, W. Hon. Sec., Miss C. Vincent. By subscriber's letter. Out-patients pay 6*d.* entrance fee, and 2*d.* each visit. | *In-Physicians*, Drs. Anderson, Atkins, Marshall, de la Cherois. *Out-Physicians*, Drs. Marshall, De la Cherois, Hitchcock. Daily, 1 to 3, and S 9 to 10. (All the physicians are women) |
| North-West London Hospital, 18, Kentish Tn. Rd., N.W | W. 1.30. *See* General Hospitals |
| Royal Free Hospital, Gray's Inn Road, W.C. | *In- and Out-Physician*, Dr. Hayes, Tu. S. 9 |
| Royal Hospital for Children and Women, Waterloo Bridge Road, S.E. Sec., Mr. R. G. Kestin. | *Out-Physicians*, Daily at 2. *Out-Surgeon*, W. 2. *See* "Children's Diseases." |
| St. Bartholomew's Hospital, Smithfield, E.C. | *In-Physician*, Dr. M. Duncan, Tu. Th. *Out-Physician*, Dr. Godson, W. S. 9 |
| St. George's Hospital, Hyde Park Corner, S.W. | *Physician*, Dr. Champneys, Th. 1.30 |
| St. Mary's Hospital, Cambridge Place, Paddington, W. | *Physicians*, Drs. Meadows, Tu. F. 9.30; H. Jones, M. Th. 1.30 |
| St. Thomas's Hospital, Westminster Bridge Road, S.E. | *Physician*, Dr. Jervis, Tu. F. 2 |
| Samaritan Free Hospital for Women and Children, Lower Seymour Street, W. Branch, 1, Dorset Street, Manchester Square, W. Sec., Mr. Geo. Scudamore. Governor's letter or card is required if the disease of the applicant is *not* peculiar to her sex. Out-patients department (free) is at Edward's Mews, Duke Street, W. | *In-Physicians*, Drs. P. Boulton, M. Prickett, Amand Routh. Daily, 2. *In-Surgeons*, Messrs. G. G. Bantock, J. H. Thornton, W. A. Meredith. Daily, 9.30. *Out-Physicians*, Drs. M. Prickett, and Amand Routh, W. S. 1.30. *Out-Surgeons*, Messrs. W. A. Meredith, and J. D. Malcolm, M. Th.; A. H. G. Doran, and W. S. A. Griffith, Tu. F. Hour 1.30 |
| University College Hospital, Gower Street, W.C. | *Physicians*, Drs. G. Hewitt, M. Th. 1.30; J. Williams, Tu. F. 2 |
| West London Hospital, Hammersmith Road, W. | *Physicians*, Drs. A. Venn, Moullin, W. S. 2 |
| Westminster Hospital, Broad Sanctuary, S W. | *Physicians*, Drs. Potter, Tu. F. 2; Grigg, Tu. F. 9 |

